# Supplementary material for: FT-IR Spectral Signature of Sensitive and Multidrug-Resistant Osteosarcoma Cell-Derived Extracellular Nanovesicles
Source: Cells. 2022 Feb 23;11(5):778. doi: 10.3390/cells11050778 (PMC8909163; doi:10.3390/cells11050778)
Supplement: Supplementary file 1 [file cells-11-00778-s001.zip › Supplementary figure Rev/Figure Captions rev.pdf]

Figure S1. FT-IR/ATR spectra of PBS.

Figure S2. Reproducibility of FT-IR curves. In the graph, two aliquots from one same sample were analyzed to assess intra-sample variability.

Figure S3. FT-IR/ATR spectra of 143B cells derived- EVs. Spectra of EVs after scaling with respect to the band at 1649 cm−1 (zooms in the protein areas)

Figure S4. MSC-derived extracellular nanovesicles (EVs) characterization. (a) Representative transmission electron microscopy images of EVs, isolated from medium conditioned by MSC cells (scale bar: 100 nm). (b) Protein content-based EVs characterization was assessed by Western blot analysis for the expression of transmembrane proteins and cytosolic protein.
