# Supplementary figures and images for: FT-IR Spectral Signature of Sensitive and Multidrug-Resistant Osteosarcoma Cell-Derived Extracellular Nanovesicles
Source: Cells. 2022 Feb 23;11(5):778. doi: 10.3390/cells11050778 (PMC8909163; doi:10.3390/cells11050778)

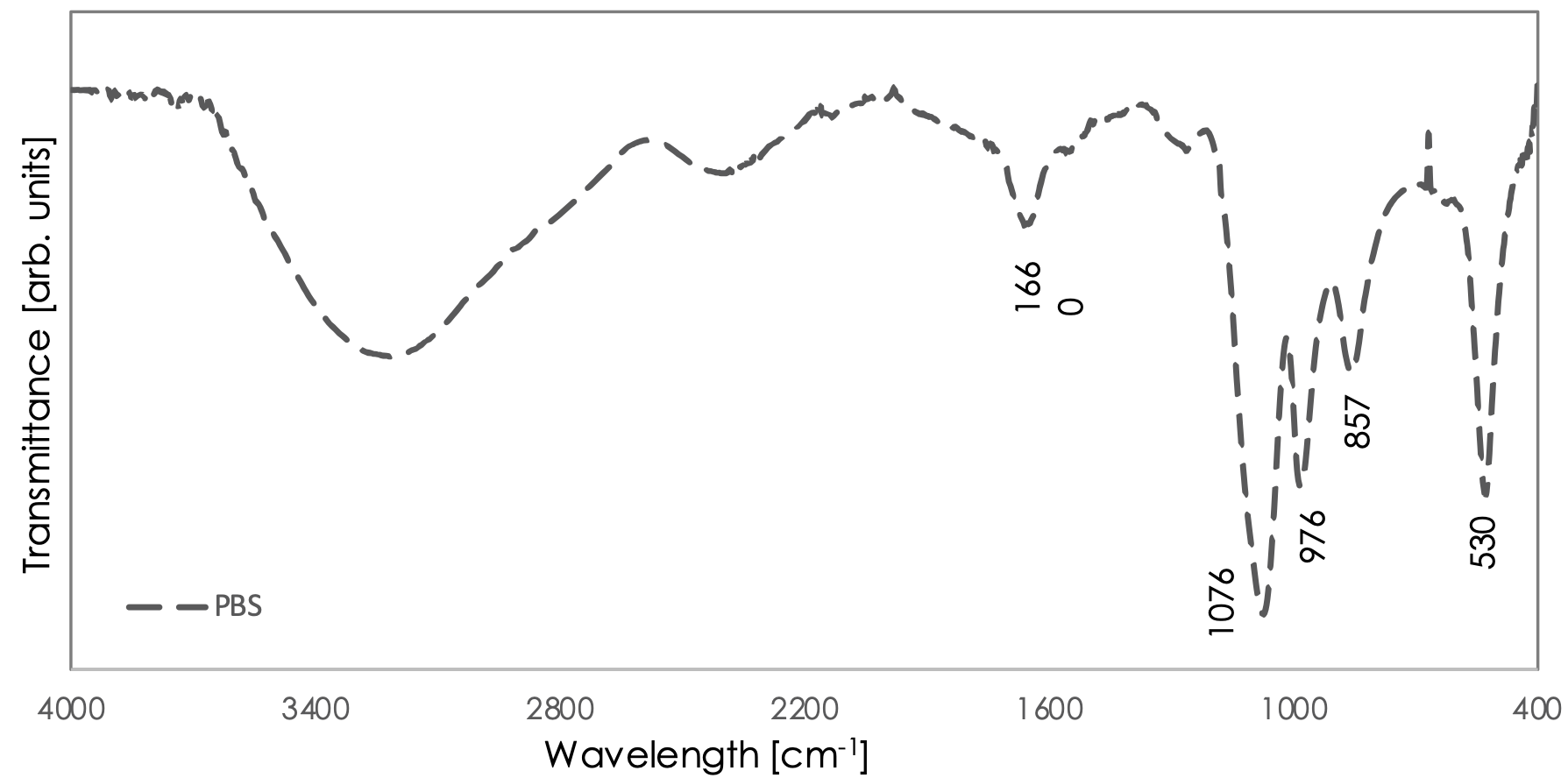

Supplement: Supplementary file 1 [file cells-11-00778-s001.zip › Supplementary figure Rev/figure S1.tiff]

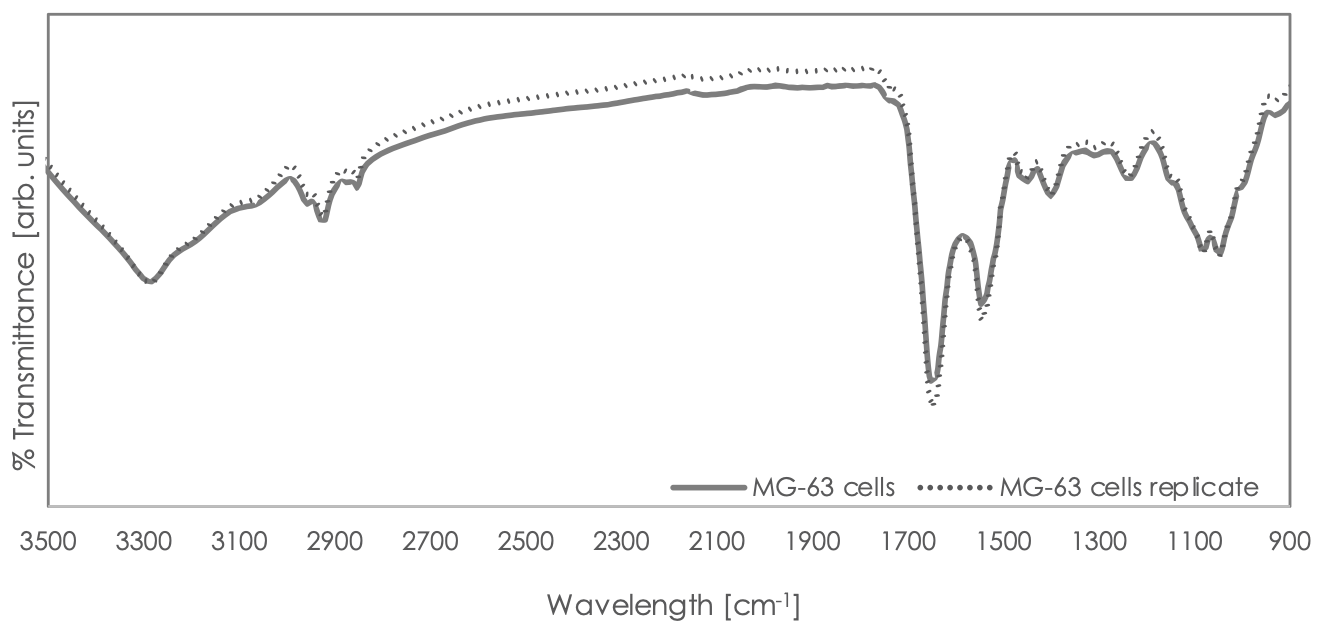

Supplement: Supplementary file 1 [file cells-11-00778-s001.zip › Supplementary figure Rev/figure S2.tiff]

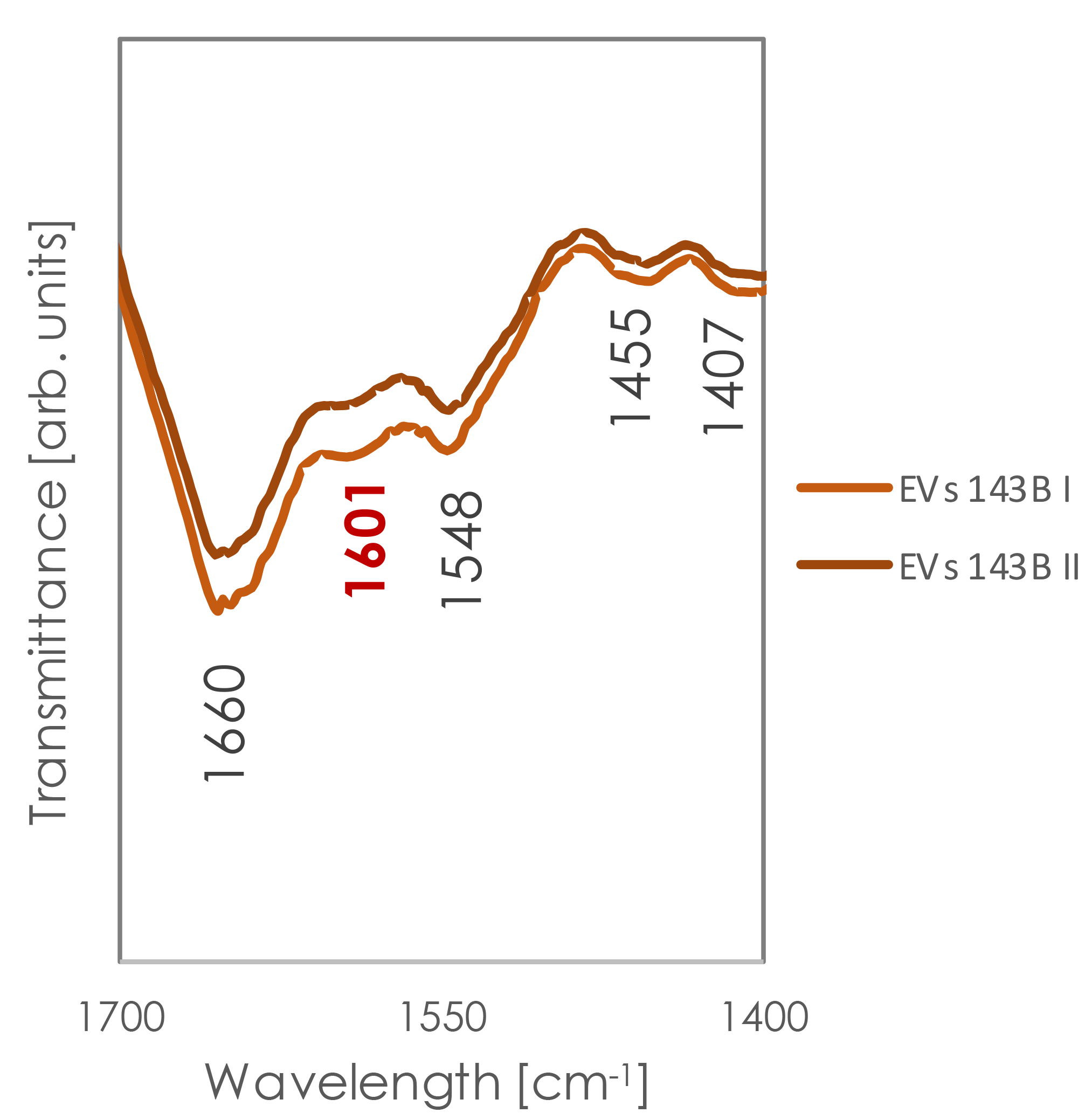

Supplement: Supplementary file 1 [file cells-11-00778-s001.zip › Supplementary figure Rev/figure S3.tif]

a

EVs MSC

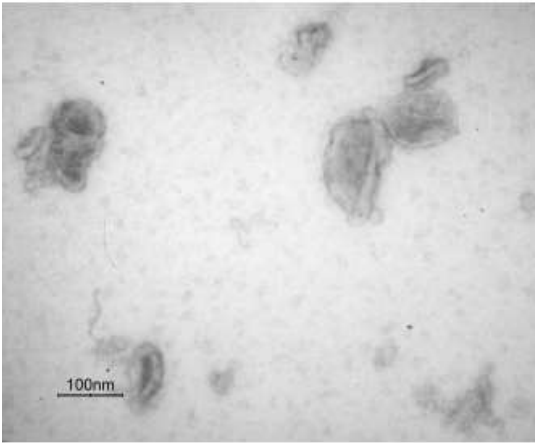

b

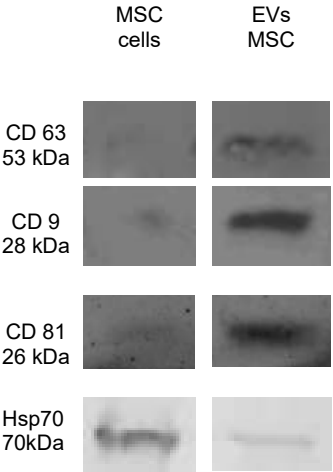

Supplement: Supplementary file 1 [file cells-11-00778-s001.zip › Supplementary figure Rev/figure S4.pdf]
